# Return on investment of self-measured blood pressure is associated with its use in preventing false diagnoses, not monitoring hypertension

**DOI:** 10.1371/journal.pone.0252701

**Published:** 2021-06-18

**Authors:** Alejandro Arrieta, John Woods, Gregory Wozniak, Stavros Tsipas, Michael Rakotz, Stephen Jay

**Affiliations:** 1 Robert Stempel College of Public Health and Social Work, Florida International University, Miami, Florida, United States of America; 2 Indiana University Richard M. Fairbanks School of Public Health, Indianapolis, Indiana, United States of America; 3 Improving Health Outcomes, American Medical Association, Chicago, Illinois, United States of America; 4 Indiana University School of Medicine, Indianapolis, Indiana, United States of America; Sao Paulo State University (UNESP), BRAZIL

## Abstract

Previous research indicates that patient self-measured blood pressure (SMBP) is a cost-effective strategy for improving hypertension (HTN) diagnosis and control. However, it is unknown which specific uses of SMBP produce the most value. Our goal is to estimate, from an insurance perspective, the return-on-investment (ROI) and net present value associated with coverage of SMBP devices when used (a) only to diagnose HTN, (b) only to select and titrate medication, (c) only to monitor HTN treatment, or (d) as a bundle with all three uses combined. We employed national sample of claims data, Framingham risk predictions, and published sensitivity-specificity values of SMBP and clinic blood-pressure measurement to extend a previously-developed local decision-analytic simulation model. We then used the extended model to determine which uses of SMBP produce the most economic value when scaled to the U.S. adult population. We found that coverage of SMBP devices yielded positive ROIs for insurers in the short-run and at lifetime horizon when the three uses of SMBP were considered together. When each use was evaluated separately, positive returns were seen when SMBP was used for diagnosis or for medication selection and titration. However, returns were negative when SMBP was used exclusively to monitor HTN treatment. When scaled to the U.S. population, adoption of SMBP would prevent nearly 16.5 million false positive HTN diagnoses, thereby improving quality of care while saving insurance plans $254 per member. A strong economic case exists for insurers to cover the cost of SMBP devices, but it matters how devices are used.

## Introduction

Hypertension (HTN) accounts for 1 in 6 deaths annually [1], at a cost of $52.4 billion to the U.S. health care system [2]. As a common chronic disease, it is screened for at nearly every primary care visit [3] and yet it remains misdiagnosed in 10 to 20% of U.S. adults [4]. Although it is controllable [5], over half of adults with hypertension remain uncontrolled [2, 6, 7].

One recommended strategy for improving diagnosis and control of HTN is to use self-measured blood-pressure (SMBP) monitoring [8]. For this analysis, we assumed patients used validated devices with proper cuff size (AMA/AHA joint website, https://www.validatebp.org/ provides an up-to-date list of validated SMBP devices). It was further assumed that patients used the devices properly according to accepted protocol. Proper use includes the following elements: (1.) patients are seated at rest for five minutes, (2.) back supported, (3.) arm supported with cuff at heart level (4.) both feet on the floor, (5.) with final BP used for interpretation consisting of the average of two BP readings in the morning and two in the evening for a period of 7 days (minimum of 3 days or 12 readings) [8, 9].

Previous studies indicate that, when properly used, SMBP monitoring is more effective than clinic blood pressure monitoring (CBPM) for diagnosing and managing hypertension [8, 10–12], especially when SMBP is used in conjunction with counseling and support [13]. SMBP also has better predictive accuracy in forecasting end-organ damage [14] and adverse cardiovascular events [15]. A recent Community Preventive Services Task Force (CPSTF) review [16] of 29 economic studies found that SMBP is also cost-effective when used with additional patient support or team-based care. In light of their review, the CPSTF identified three evidence gaps requiring further study: (1.) studies that distinguish between the various uses of SMBP in order to better understand how each use contributes separately to its economic value; (2.) studies that evaluate return on investment (ROI) and long-term changes in morbidity and mortality associated with the stand-alone use of SMBP; and (3.) studies that evaluate incremental change in cost-effectiveness as various levels of support and counseling are added to stand-alone SMBP. The current study helps close the first two of these gaps by simulating the economic benefit associated with three different uses of SMBP and their long-term impact on morbidity and mortality from the perspective of the insurer.

We build on a previous decision-analytic simulation model, which was developed to estimate the ROI of SMBP for a large midwestern self-insured employer health plan [17]. The extended model estimates ROI to the scale of the U.S. adult population and separates the economic contributions of three distinct uses of SMBP: (a) for diagnosing HTN, (b) for selecting and titrating medication, and (c) for monitoring HTN treatment. Our objective was to determine, using nationally representative parameters, which of these clinical uses has the greatest economic value from the perspective of a private insurer.

Our results indicate that a strong business case exists for insurers to cover the cost of SMBP devices, but it matters how the devices are used. Although SMBP is most often used as a tool to monitor blood pressure (BP) in those under treatment for HTN, we find that the primary economic value of SMBP from an insurance perspective comes from its use in diagnosing new-onset HTN and in selecting and titrating medication, not from ongoing treatment monitoring.

## Methods

### Interventions and use cases

We compared two interventions: (a) CBPM (usual care): hypertension diagnosed and managed based solely on episodic CBPM with no additional monitoring outside the clinic, and (b) SMBP (augmented care): SMBP complements CBPM. These two interventions were compared with respect to three use cases:

1. Diagnosis only: CBPM and SMBP compared when used solely to *diagnose* HTN. The hypothesized benefit of SMBP in this case is the dollars saved by the insurer by preventing unnecessary prescriptions and fewer adverse drug events as a result of the greater diagnostic accuracy of SMBP.
2. Treatment selection and titration only: CBPM and SMBP compared when used solely to *select and titrate treatment* (but not to establish the diagnosis). In this case, the diagnosis would have been established previously with either CBPM or SMBP. The hypothesized benefit of SMBP is the dollars saved by the insurer by preventing unnecessary office visits as a result of more efficient treatment selection and dosage titration when SMBP is used [18, 19].
3. Ongoing HTN treatment monitoring: CBPM and SMBP compared when used solely for *ongoing HTN treatment management and BP control* (but not to diagnose HTN or to select and titrate treatment). The hypothesized benefit in this case is the dollars saved from better patient adherence to treatment and better BP control with SMBP [14, 20, 21], which translates into lower costs due to fewer downstream hypertension-related cardiovascular disease (CVD) events [22, 23].

### Modeling approach

We use a decision-analytic simulation model consisting of two parts, a decision tree and a Markov model. The decision-tree (see Fig A in S1 Appendix) accounts for information an insurer would have about a cohort of new enrollees entering a health insurance plan, i.e., medical histories and HTN diagnosis based on CMBP. Entry into the Markov process begins in year two (See Fig B in S1 Appendix), and accounts for the transition of hypertensive health-plan members through various CVD states—stroke, myocardial infarction (MI), or other CVD events. Those who are normotensive are considered to be free of excess hypertension-related risk (~CVD).

Compared to our previous model [17], we extend the ROI estimation to the scale of the U.S. adult population by: (a) using Framingham equations [24] to predict 10-year CVD risk rather than relying on local claims data from a single self-insured employer to calculate 4-year risk; (b) using parameters representative of the U.S. population, rather than relying on local claims data for those parameters, and; (c) employing a larger, national sample of claims from a database of 4,002,866 covered lives, of whom 1,621,496 (41%) had a diagnosis of hypertension, rather than relying on a smaller self-insured employer database. We re-specified the extended model so that the three SMBP applications could be evaluated separately to determine how its economic value varies depending on usage. The resulting simulations are generalizable to the entire U.S. adult population, capture longer-term risk of CVD-related events, and provide a more detailed understanding of the economic benefits of specific SMBP usages from an insurer perspective.

### Data sources and parameters

The probabilities governing the decision-tree and Markov model are presented in the S1 Appendix with referenced sources. Sensitivity and specificity values (Table B in S1 Appendix) for SMBP and CBPM were obtained from estimates reported by Lovibond, et al. [25] based on a previous meta-analysis of Hodgkinson, et al. [26]. The frequency of office visits during the treatment-selection period (Table D in S1 Appendix) was obtained from the Truven Health MarketScan^®^ Lab Database, which provides a national sample of commercial insurance claims [27].

Treatment adherence rates, patient characteristics, age-related diagnostic history, and HTN prevalence (Table B in S1 Appendix) were obtained from National Health and Nutrition Examination Survey (NHANES) 2013–2014 data [28]. Ten-year Framingham risk equations [24] were used to estimate age-dependent probabilities associated with annual transitions from non-CVD to CVD states among individuals diagnosed with HTN (Table C in S1 Appendix). CVD event probabilities were obtained for each year as the cohort aged to a maximum age of 100, at which point all individuals were assumed to have left the health plan or died. Details of all parameter estimates are provided in the S1 Appendix. This study is exempt of ethical review because it uses public deidentified secondary data that does not involve "human subjects" (as defined by federal regulations and guidance).

### Intervention costs, medical savings, and health insurance parameters

Annual per-patient baseline costs for managing HTN were estimated at $875 for those adhering to treatment, and $528 for those not adhering [29]. First-year per-patient costs of a CVD event were estimated at $57,243 for a MI, $47,997 for a stroke, and $22,156 for other CVD events [30], which included unstable angina and transient ischemic attack. Costs of post-CVD states converge to baseline after 5 years. Estimated intervention costs, insurance premium estimates and cost-sharing arrangements (deductibles and copayments) were converted to 2019 U.S. dollars. All values and sources are provided in detail in Tables D and E in the S1 Appendix.

### ROI and NPV of SMBP monitoring

Differences from CBPM were calculated to obtain net costs, net savings, and net benefits (net savings minus net costs) associated with SMBP. Incremental ROIs for SMBP relative to CBPM were calculated as the ratio of net benefits divided by net costs.

NPV is the net dollar-benefit of SMBP compared to CBPM, expressed as the difference in savings minus the difference in cost between SMBP and CBPM converted to 2019 U.S. dollars. An annual 3% discount rate was applied to all dollar-costs and benefits.

### Uncertainty and sensitivity analysis

Monte Carlo simulations were performed to account for uncertainty in parameter estimates (i.e., CVD transition probabilities, prevalence and utilization rates, health care costs and insurance cost-sharing). Age-specific probabilities of a favorable NPV were defined as the fraction of Monte Carlo samples that produced a positive NPV. We calculated overall NPV, as well as NPVs for three use cases (i.e., diagnosis only, treatment selection and titration only, or HTN treatment monitoring only). Sensitivity analyses were performed to explore changes in NPV associated with variations in baseline assumptions related to diagnostic accuracy (SMBP measurement specificity), the treatment-selection-titration process (number of office visits), and ongoing BP monitoring (adherence to treatment and BP reduction among those diagnosed with HTN). Results of sensitivity analyses are provided in the S1 Appendix.

## Results

### Financial impact of SMBP monitoring

Table 1 shows the financial impacts associated with insurance coverage of SMBP devices at 1- and 3-year time horizons, and as a lifetime total for all use cases considered as a bundle. ROIs and NPVs are time- and age-sensitive. The economic benefit is largest in the first year and in the youngest age groups (first-year ROI is 499% or $49.90 per dollar invested for those aged 25–34, but only 36% for those 75–84. Only in age-group 85+ is the ROI negative, i.e., -64% or -$6.40 per dollar invested).

**Table 1:** Financial impact of SMBP (ROI, NPV) from the perspective of a private insurer. Estimates are for all use cases bundled together.

Age Group	Return on Investment (ROI) ^a^ (average per individual)			Net Present Value (NPV) ^b^ (average per individual)		
	1-Year ROI ^c^	3-year ROI ^d^	Lifetime ROI ^e^	1-Year NPV ^c^	3-Year NPV ^d^	Lifetime NPV ^e^
Age 25–34	499%	479%	470%	$322	$395	$442
Age 35–44	451%	430%	422%	$280	$342	$379
Age 45–54	365%	339%	330%	$227	$278	$309
Age 55–64	163%	139%	130%	$105	$123	$135
Age 65–74	79%	59%	53%	$50	$53	$56
Age 75–84	36%	20%	15%	$22	$17	$16
Age 85+	-64%	-72%	-75%	-$37	-$60	-$70
**Total Per Individual**				**$190**	**$229**	**$254**

^a^ ROIs have the following meaning. If $1.00 is invested (i.e., goes to reimburse SMBP) and the investment returns that dollar plus an additional $1.00 in savings, the ROI would be 100% (a doubling of the original amount invested). An ROI of 0% means the amount invested equals the amount returned (a breakeven investment). A negative ROI means the amount invested exceeds the amount returned (an investment loss).

^b^ An NPV of $100 means the present-day value of the investment (i.e., insurance coverage for the SMBP device) is $100 more than the amount originally invested. A negative NPV means the present-day value is worth less than the amount invested (an investment loss). All NPVs expressed in 2019 dollars.

^c^ A 1-year ROI and NPV capture all costs and benefits produced in the first year of the investment of SMBP devices.

^d^ A 3-year ROI and NPV capture all costs and benefits produced for the first five years of the investment of SMBP devices.

^e^ A lifetime ROI and NPV capture all costs and benefits produced over the lifetime of the cohort under analysis. Notice that due to the insurance turnover, our lifetime definition also includes members who are alive but leave the insurance plan every year.

Table 2 presents NPVs by source of savings and by SMBP use case. Coverage of SMBP monitors is negative (-$127 per individual) since this represents a cost. Costs are offset by savings, expressed as NPVs associated with: (a) hypertension diagnosis, treatment selection and dosage titration (saving $210 per individual when SMBP is used), (b) ongoing HTN treatment monitoring (saving $202 per individual), (c) treatment of future hypertension-related CVDs (saving <$1 per individual), and (d) premiums paid to the insurance plan by surviving plan members (saving <$1 per individual).

**Table 2:** Financial impact of SMBP by origin of savings and by use case (hypertension diagnosis only, treatment selection and titration only, or management only).

Age Group	Breakdown of lifetime NPV ^a^ by origin of savings (per individual)					Lifetime NPV ^a^ by SMBP use case (per individual)		
	SMBP Device Investment	Hypertension Diagnosis, Treatment Selection, and titration	Ongoing Hypertension Management after Diagnosis	Treatment of Future Hypertension-Related CVDs	Insurance Premium	When Used for Diagnosis Only	When Used for Treatment Selection Only	When Used for Ongoing BP Monitoring Only
Age 25–34	-$125	$327	$306	$0	$0	$264	$25	-$120
Age 35–44	-$115	$285	$257	$0	$0	$221	$22	-$110
Age 45–54	-$123	$238	$235	$0	$0	$204	$18	-$118
Age 55–64	-$141	$139	$148	$1	$0	$127	$12	-$136
Age 65–74	-$140	$93	$98	$3	$0	$81	$9	-$129
Age 75–84	-$126	$71	$64	$1	$0	$49	$7	-$118
Age 85+	-$106	$21	$9	-$2	-$1	$5	$3	-$108
**Total Per Individual**	**-$127**	**$210**	**$202**	**$0**	**$0**	**$173**	**$17**	**-$121**

^a^ A lifetime NPV captures all costs and benefits produced over the lifetime of the cohort under analysis. Notice that due to the insurance turnover, our lifetime definition also includes members who are alive but leave the insurance plan every year. All NPVs expressed in 2019 dollars.

The last three columns in Table 2 show NPVs broken down by SMBP usage. NPVs are positive when all use cases are bundled. When uses are assessed one-by-one, savings are realized when SMBP is used to diagnose new onset HTN (Use Case #1: NPV = $173 per individual) or to select and titrate treatment (Use Case #2: NPV = $17 per individual). Investment losses are incurred when SMBP is used solely to monitor HTN treatment (Use Case #3: NPV = -$121 per-individual). The positive NPVs associated with the first two uses are a result of SMBP’s superior diagnostic specificity and greater efficiency (fewer office visits required) in selecting and titrating medications.

### Health impact of SMBP

The financial impact of SMBP is driven in part by its health effects shown in Table 3. The most noteworthy effect of SMBP is its capacity to reduce false-positive (FP) diagnoses. SMBP would prevent a FP diagnosis in about 420 individuals, which would imply the prevention of unnecessary treatment of 221 individuals in a population of 1,000. When scaled to the U.S. population, adoption of SMBP would prevent nearly 16.5 million FP HTN diagnoses, and 8.2 million unnecessary treatments. Had CBPM been used instead, these individuals would have been falsely diagnosed as hypertensive and exposed to the costs and risks associated with lifelong medication usage. This is a direct effect of the greater diagnostic specificity of SMBP.

**Table 3:** Health impact of SMBP (changes in treatment, morbidity, and mortality).

Age Group	Changes in Hypertension Treatment Resulting from SMBP Use (per 1,000 population)			Changes in CVD Morbidity and Mortality Resulting from SMBP Use (per 100,000 population)	
	Additional False Positive Hypertension Diagnosis	Additional Normotensive Individuals Who Would Avoid Receiving Unnecessary Treatment	Additional Hypertensive Individuals Who Would Receive Needed Treatment	Expected Changes in Non-fatal CVD Events	Expected Changes in Deaths at 10 Years
Age 25–34	-114.7	32.0	0.7	57.3	0.0
Age 35–44	-104.5	45.9	1.2	78.6	0.0
Age 45–54	-87.6	52.6	1.1	114.9	0.1
Age 55–64	-48.7	36.5	2.5	-278.0	0.2
Age 65–74	-32.6	28.1	1.9	-1,034.0	-0.4
Age 75–84	-24.6	20.8	3.1	-153.4	0.4
Age 85+	-6.7	4.8	4.8	151.7	2.3
**Total**	**-419.6**	**220.7**	**15.4**	**-1,062.9**	**2.5**

In a population of 1,000 individuals, SMBP would also put nearly 15.4 additional hypertensive individuals under treatment, individuals who would have gone undiagnosed and untreated had CBPM been used. This effect is attributable to SMBP having slightly better sensitivity than CBPM.

Finally, SMBP has a long-term morbidity impact, resulting in an average 1,063 fewer non-fatal CVD events per 100,000. This reduction occurs mainly in older age groups. SMBP has virtually no effect on predicted 10-year mortality.

## Conclusions

The critical new message that this decision-analytic simulation model conveys is that the economic benefits of SMBP from an insurance perspective depend on how SMBP is used. In light of the recent Community Preventive Services Task Force (CPSTF) challenge calling for studies that separate the various uses of SMBP [16], our study finds that the economic value of SMBP from an insurance perspective comes from its use in diagnosing HTN (Use Case #1), or selecting and titrating treatment (Use Case #2). When SMBP is used only for ongoing HTN treatment monitoring (Use Case #3), the NPV is negative for all age groups (Table 2).

The CPSTF review also called for studies that evaluate ROI and long-term changes in morbidity and mortality associated with the stand-alone use of SMBP. We find that positive returns to the insurer occur quickly, within the first year, and although the economic value of SMBP is larger for younger individuals, it remains positive for all age groups up to age 85 (Table 1). This short-term positive ROI should partially alleviate insurers’ concerns regarding the possibility of investment loss due to annual member turnover rates. The long-term impact of SMBP monitoring on CVD morbidity and mortality is small, which comports with the finding that the primary economic value of SMBP is its greater specificity in ruling out false diagnoses and avoiding the lifelong costs of medications and their associated adverse effects [31].

We deliberately adopted an insurance perspective rather than a societal perspective to determine whether there exists a private-market business rationale for reimbursing the cost of SMBP devices. This perspective is relevant in the market-oriented U.S. healthcare system, where lack of reimbursement by insurance plans continues to be a barrier to widespread use of SMBP. There are a few recent examples of plans covering SMBP devices. The Blue Cross and Blue Shield Government-wide Service Benefit Plan, Federal Employee Program^®^ (FEP^®^), through its *Hypertension Management Program*, provides devices as a covered benefit to eligible enrollees [32]. While coverage for SMBP devices is not mandated in Medicare Part C, those plans may include benefits for in-home equipment [33]. Individuals with flexible spending accounts and enrollees in high-deductible plans with health savings accounts may also pay for SMBP devices from those accounts. Additionally, two new provider CPT codes are available as of January 2020 for reporting SMBP monitoring [34].

Our results for the U.S. adult population combining all SMBP Use Cases are consistent with previous studies that have largely supported the idea that SMBP is cost-effective compared to CBPM [16, 17, 35]. In those studies, all SMBP Use Cases (diagnosis, treatment selection/titration, and BP monitoring) are usually considered as a bundle. Our study suggests that the cost-effectiveness of SMBP is driven by the positive economic benefits of Use Cases #1 and #2, which compensate for losses incurred in Use Case #3.

In a similar study to ours, Beyhaghi and Viera (B-V) [36] estimated the cost-effectiveness of SMBP Use Case #1 (hypertension diagnosis). These authors found that SMBP was not cost-effective compared to CBPM. The B-V model is similar to ours in many respects—both models take an insurer perspective, and both use data from NHANES and Framingham [24] to estimate risk factors and CVD incidence rates. However, the major difference that explains the discrepant results is that B-V used sensitivity and specificity values for SMBP and CBPM from Hodgkinson et al. [26], whereas we used estimates from Lovibond et al. [25] (which were derived from, but are not identical to, Hodgkinson). As shown in our sensitivity analysis (Table F in S1 Appendix), investment in SMBP monitoring is highly sensitive to the accuracy of SMBP measurement in detecting white-coat hypertension (WCH). A 10% reduction in SMBP specificity reduces the investment’s NPV by more than 45%, with higher reductions among older age groups. While evidence has largely supported the idea that SMBP is cost-effective compared to CBPM [16, 35], more research is needed to assess sensitivity and specificity values to SMBP under real life conditions.

Our modeling approach has some limitations. Although we show that payers who reimburse the cost of SMBP devices can expect to see positive economic returns, our model does not directly address the implications of how a large-scale change in SMBP reimbursement policy might affect provider workload and utilization, and how that might alter the overall cost-benefit balance. If major national insurers began to cover SMBP and if this led to an expansion in its use, payments to physicians would increase, reflecting the influx of greater numbers of patients using SMPB devices. However, as our model illustrates, not only the cost, but also the benefits of SMBP monitoring would expand under that scenario, resulting in further net savings. More research is needed to fully understand how the cost-benefit calculus might change with large-scale SMBP adoption.

We modeled what an insurance plan would know about HTN prevalence rates among a cross-section of age-specific cohorts. We did not model longitudinal BP changes that providers would see as they monitor individual patients. Since the greater diagnostic accuracy of SMBP would be expected to confer greater precision in detecting incremental BP changes over time, our model almost certainly undervalues the full economic benefit of SMBP as a diagnostic tool for detecting small rises in BP that eventually may lead to new-onset HTN as patients age.

The ability of SMBP to better identify patients with WCH and, therefore, to prevent these individuals from being exposed to the costs and risks of unnecessary treatment, assumes that WCH is a benign condition, an assumption that is currently being debated [37]. Several earlier studies showed that neither untreated WCH nor treated white-coat effect (WCE) were associated with increased risk of CVD events [38, 39], and both the 2011 U.K. National Institute of Health and Clinical Excellence guideline [40] and the 2015 U.S. Preventive Services Task Force guideline [41], recommend out-of-office BP monitoring to confirm that the patient does not have WCH *before* initiating antihypertensive medication. However, more recent reviews [42–44] have concluded that WCH, but not WCE, is associated with a slightly elevated CVD risk compared to normotensive individuals (although the risk is substantially lower than that for sustained HTN). This implies that those identified as having WCH, especially older individuals with other CVD risk factors [45], may not be risk free and should be followed with SMBP to determine if they are developing sustained HTN requiring treatment. Our model captures the savings from SMBP associated with correctly identifying, and therefore not over-treating those with WCH, but it does not capture the small although perhaps not inconsequential savings associated with identifying and, consequently not under-treating those who are transitioning from untreated WCH to sustained HTN.

Consistent with the insurance perspective, we did not account for provider co-interventions (i.e., device validation, SMBP data interpretation and management, staff education, or patient lifestyle counseling), which is probably necessary for self-monitoring to produce clinically-meaningful BP reductions large enough to have a long-term economic impact. A recent patient-level meta-analysis of 7,138 patients concluded that self-monitoring alone does not significantly lower BP or lead to better control unless it is provided in conjunction with co-interventions [13]. This is consistent with our model showing that stand-alone SMBP monitoring is not associated with BP changes in treated HTN large enough to produce a positive ROI for insurers.

Insurers have been slow to provide reimbursement for SMBP devices, presumably because they remain skeptical that coverage would yield positive economic returns. Our model demonstrates that a positive business case exists, but it matters how SMBP is used. In contrast to most clinical programs that emphasize the use of SMBP as a tool for monitoring BP in those under treatment for HTN, our model indicates that its greatest *economic* value is actually associated, not with the monitoring of HTN treatment, but with HTN diagnosis and treatment selection and titration. ROIs were negative only when SMBP was used solely to monitor BP in patients being treated for HTN. This may be a primary reason why insurers are hesitant to offer coverage. From the perspective of an insurer who views SMBP as a treatment management tool, the likelihood that long-term BP monitoring will produce economic benefits by averting future myocardial infarctions and stroke may seem far off and uncertain. However, the immediate economic benefits associated with improved diagnosis and medication titration, as quantified by the present model, provide a realistic business rationale for insurers to consider in deciding to cover SMBP devices. Given our finding that SMBP maintains its value under a bundled-usage scenario, we conclude that a rational practice would be to implement SMBP for all three uses—diagnosis, treatment selection and titration, and HTN treatment monitoring.

## Supporting information

S1 Appendix Expanded methods. (PDF)
