# Supplementary material for: Return on investment of self-measured blood pressure is associated with its use in preventing false diagnoses, not monitoring hypertension
Source: PLoS One. 2021 Jun 18;16(6):e0252701. doi: 10.1371/journal.pone.0252701 (PMC8213192; doi:10.1371/journal.pone.0252701)
Supplement: S1 Appendix — (PDF) [file pone.0252701.s001.pdf]

S1 Appendix

Expanded Methods

Return on Investment of Self-Measured Blood Pressure Is Associated with Its Use in Diagnosing, not Monitoring Hypertension

This study uses a decision-analytic model with two parts to describe the outcomes and costs of a hypothetical cohort of insured individuals. Two interventions are modeled and compared: self-measured blood pressure (SMBP) monitoring with SMBP measurements used in the diagnosis, treatment selection and ongoing BP monitoring (management of hypertension), and clinic blood pressure measurement (CBPM) where the usual (currently applied standard) care is based on the CBPM protocol for diagnosis and treatment selection, and no additional support for management of hypertension.

1. Model

The model consists of two primary parts: Part 1, characterized by a decision tree model, describes the diagnosis, treatment selection and management decision process. Part 2, characterized by a Markov model, describes the transition to different cardiovascular disease (CVD) events (stroke, myocardial infarction or other CVD events). Seven age groups are modeled: 25 to 34, 35 to 44, 45 to 54, 55 to 64, 65 to 74, 75 to 84, and 85 or more years old. Individuals within each age group are assumed to have the same health characteristics and to be insured by a commercial private insurance. All insured individuals go through part 1 of the model in year 1, and then move and remain in part 2 of the model until they die or leave the health insurance plan. The model assumes annual cycles. All costs and returns are in 2016 US dollars and were discounted at a 3% annual discount rate. All simulations were performed in Microsoft Excel.

1.1 Decision tree model

Fig A describes the first part of the model.

(a) In the first year, a hypothetical cohort of individuals can take one of three pathways according to their previous history of blood pressure (BP) screening and hypertension diagnosis based on usual care (see probability of diagnosis in Table A).

<u>Pathway 1</u>: Individuals previously screened and given a diagnosis of hypertension (DxHT). Based on known CBPM sensitivity and specificity (1), we assumed that a fraction of these individuals, including those with white-coat hypertension (WCH) who exhibit high blood pressure in the clinic but not outside the clinic (2), would be diagnosed incorrectly and we classified them as false positives (FP). The remaining fraction was assumed to be correctly diagnosed and were classified as true positives (TP). The model mirrors usual practice by

assuming that these individuals are not re-evaluated with SMBP, since this would necessitate taking them off medications. The model also assumes that some of those classified as hypertensive (whether or not the diagnosis was correct) would adhere to recommended treatment (Tx) and some would not (~Tx) (3, 4).

Pathway 2: Individuals previously screened and determined to be normotensive (DxNT). A fraction of these, including individuals with masked hypertension whose BP is normal when measured in the clinic but high outside the clinic (5, 6), were assumed to be incorrectly diagnosed and were classified as false negatives (FN). The remaining fraction of normotensive individuals were assumed to be correctly diagnosed and were classified as true negatives (TN). We assumed these individuals would be re-evaluated with SMBP and those confirmed to be normotensive would not receive treatment.

Pathway 3: Individuals with no screening or diagnostic history and whose diagnosis is unknown (~Dx). We assumed these individuals would be evaluated with SMBP.

(b) The true prevalence of hypertension is unobserved and no intervention is perfectly accurate in detecting hypertension. Among those diagnosed as hypertensive, we assumed, based on published sensitivity and specificity estimates for SMBP or usual care via CBPM (1), that a fraction was incorrectly diagnosed (false positives, FP) and the remainder were correctly diagnosed (true positives, TP). Similarly, among those diagnosed as normotensive, we assumed a fraction was incorrectly diagnosed (false negatives, FN), and the remainder were accurately diagnosed (true negatives, TN). See Table A for diagnosis accuracy rates.

(c) The model assumes that those who are normotensive (DxNT) or whose diagnosis is unknown (~Dx) would be diagnosed based on either SMBP or CBPM and be classified as hypertensive (DxHT) or normotensive (DxNT). However, those already diagnosed with hypertension (DxHT) based on CBPM would not have their diagnosis changed, since this would involve taking them off medication; therefore, these individuals would not be re-classified based on the SMBP intervention.

(d) After diagnosis, all individuals classified as hypertensives (DxHT) are offered anti-hypertensive medication regardless of their true hypertension status. A fraction of those (see Table A) adhere to the selected medication treatment (Tx), while the rest do not adhere (~Tx).

(e) Normotensive individuals (DxNT) do not receive anti-hypertensive medication (~Tx), regardless of their true hypertension status.

(f) Regardless of their diagnosis (DxHT or DxNT) and adherence to medication (Tx or ~Tx), all individuals who are truly hypertensives (TP and FN) are at risk of developing a hypertension-related CVD event, and therefore, at the end of the first year, they will move to the second part of the model (Markov).

Fig A. Annotated decision tree model.

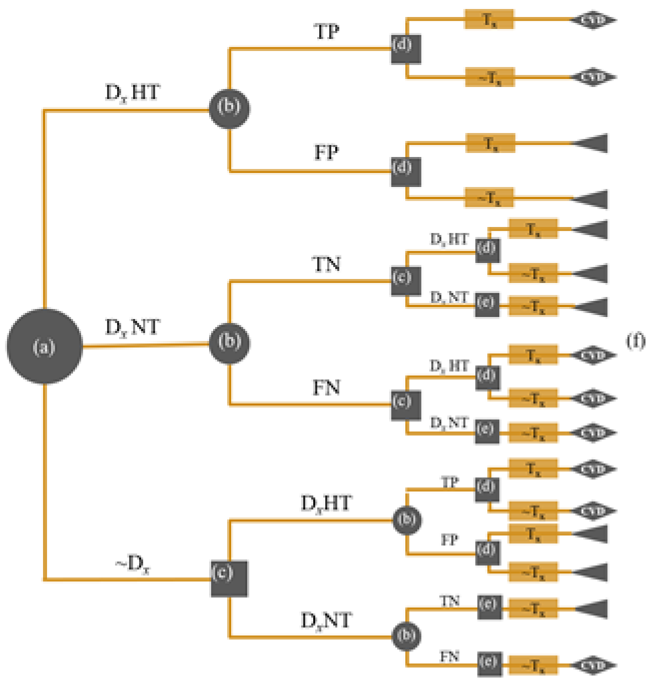

1.2 Markov model

Fig B describes the second part of the model.

Fig B. Annotated Markov model.

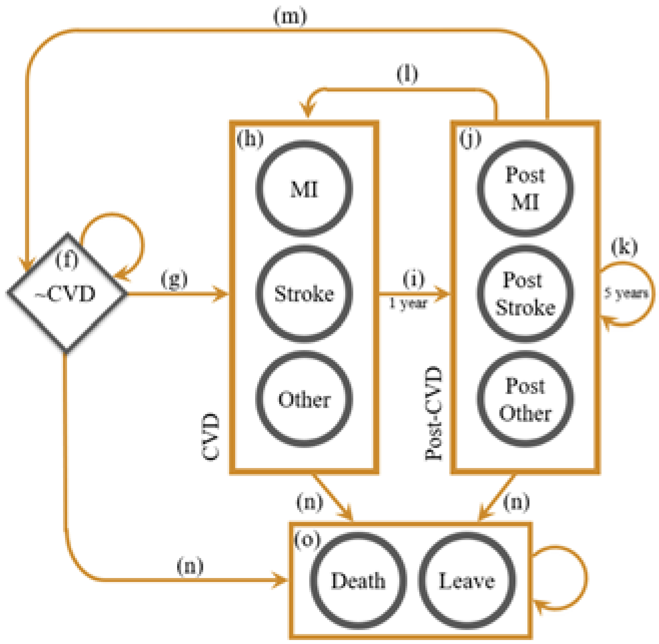

(f) All individuals who are truly hypertensive (TP and FN) start the second year in the Markov model free of CVD events (~CVD). In that year and all subsequent years, a fraction of individuals will suffer a CVD event, a fraction will die or leave the insurance plan, and therefore, exit the model permanently, and a fraction will continue to the following year free of hypertension-related CVD risk.

(g) The fraction of individuals who transit to a CVD event state will do so with a probability based on their Framingham-based CVD (stroke, myocardial infarction or other CVD) incidence rate.

(h) The CVD state represents three CVD events: stroke, myocardial infarction or other CVD event. All individuals will remain in this state for one year until they move to a post-CVD state or they exit the model due to a death event or leaving the insurance plan.

(i) Transit from a CVD to a post-CVD state occurs in one year for all surviving individuals to reflect the changes in their risk factors and costs after a CVD event.

(j) The post-CVD state represents the long-term recuperation period for stroke, myocardial infarction or other CVD events. Individuals will stay in this state for up to 5 years. However, during that 5-year period, they can suffer a subsequent CVD event, in which case they re-cycle, or they can exit the model.

(k) Individuals in the post-CVD state remain there for up to five years to reflect the time it takes to converge to baseline costs and health status at the ~CVD state.

(l) A fraction of individuals will suffer other CVD event within the first five years after their first CVD event. Subsequent events occur with an age-dependent incidence probability of a stroke, myocardial infarction or other CVD event.

(m) After five years in the post-CVD state, individuals who do not suffer a subsequent CVD event return to a baseline state of CVD risk.

(n) At any point in time, any individual can exit the model by i) dying from a non-CVD related event, ii) dying after a CVD event, or iii) leaving the health insurance plan. Both, death and leaving the plan are absorbing states, meaning that individuals in those states leave the model permanently.

2. Interventions and use cases

We model and compare two interventions: the SMBP intervention and usual care. For the SMBP intervention we assume patients receive a clinically validated SMBP device and educational support for its appropriate use. We also assume that physicians follow recommended

procedures for SMBP-based diagnosis and treatment selection (7). For BP monitoring (management of hypertension), we assume that all patients with hypertension keep the SMBP device. We assume further that the device is replaced every 5 years, and educational support regarding its use is provided annually. For usual care, we assume that hypertension is diagnosed using CBPM, with no additional BP measurements outside the clinic.

We distinguish three different use cases for SMBP:

i) Diagnosis: Use of SMBP to establish the diagnosis of hypertension. This use applies only to individuals seen by a doctor within the previous two years and found to be normotensive, and to individuals whose diagnosis is unknown. Individuals whose diagnosis was previously established using CBPM were excluded from this case, since we assumed that these individuals would not be taken off medication and re-evaluated with SMBP

ii) Treatment selection and titration: Use of SMBP to select an appropriate anti-hypertensive treatment and titrate the dosage. We assumed that physicians requiring four or more visits to select and titrate treatment using CBPM, would need one fewer visit if they used SMBP. This assumption was based on an episode-of-care analysis of claims data (see below for details) where we found that 90% of the treatment-selection-and-titration episodes required 4 or fewer office visits when CBPM was used. Our model assumes that SMBP would reduce excessive visits among the remaining 10% that require more than 4 visits. We performed a sensitivity analysis for this assumption.

iii) BP Monitoring: Use of SMBP for ongoing management of hypertension for BP control following treatment selection. In this case, the diagnosis would have been established previously, with either CMBP or SMBP, the treatment already would have been selected, and patients would now be receiving ongoing monitoring to control their BP.

For the SMBP intervention, we include all three use cases. For usual care, we do not include an out-of-clinic intervention for management of hypertension.

Device accuracies (sensitivity and specificity) and the effects of SMBP on hypertension management were obtained from the literature. We assumed that SMBP is able to improve medication adherence and reduce systolic BP (8, 9).

Table A. Effect on BP control parameters

Input Variable	Parameter	Uncertainty Range	Reference
Effect on BP control			
SMBP – Systolic BP reduction (mmHg)	-2.63	1.61 (SE)	Fletcher et al (9)
SMBP – Improved med. adherence (%)	0.21	0.13 (SE)	Agarwal et al (8)

NU: No uncertainty included.
SE: Standard error.

Episode of care analysis

We use an empirical episode-of-care analysis to characterize the diagnosis and anti-hypertensive treatment selection use cases. Data for these analysis come from the Truven Health MarketScan® Lab Database (10) on commercial insurance claims, 2009 to 2015. Because CBPM is still extensively used in the U.S., we assume that the Truven database represents usual care (diagnosis and monitoring by CBPM). We first identify patients newly diagnosed with essential hypertension based on diagnosis codes, procedure codes, and place of service. An episode of hypertensive care is then identified by considering only patients who had at least 6 months continuous enrollment before the initial hypertension diagnosis date, and at least 18 months continuous enrollment after that date. Individuals are excluded if they had an antihypertensive medication fill during the 6 months prior to the hypertension diagnosis date, since individuals who were already taking antihypertensive medications were likely diagnosed at an earlier time. The remaining patients are included in the population of newly diagnosed hypertension. Antihypertension medication fills are defined as Diuretics, Angiotensin Converting Enzyme (ACE) Inhibitors, Angiotensin Receptor Blockers (ARBs), Beta Blockers, and Calcium Channel Blockers (CCBs).

To identify the end date of an episode of care, we employ a concept of treatment equilibrium. A newly diagnosed patient is assumed to begin an episode of care in a state of disequilibrium (uncontrolled BP). To reach a state of treatment equilibrium (controlled hypertension), the physician must find a medication type and dosage combination that controls the patient's BP without causing adverse drug side effects. The episode of care begins with a new hypertension diagnosis and ends at the point in time following the diagnosis date when the patient is on a stable treatment regimen, i.e., continuously prescribed medication(s). We define a stable regimen, or treatment equilibrium, as four consecutive fills of the same antihypertensive medication and dose, with the fourth prescription fill coming 90 days following the initial hypertension diagnosis date. To determine equilibrium using claims data, we evaluate each medication fill in terms of standardized molecule and dosage per day, based on medication dosage, days' supply, and quantity dispensed. Applying this definition of treatment equilibrium, we determined how many days and how many visits each newly diagnosed patient took to reach BP control, the point of equilibrium, under a usual-care scenario. Not every patient will reach treatment equilibrium.

3. Data Sources and parameters

3.1 Part 1: Decision tree model

Following the annotations in Fig A, the following parameters were assumed in the model.

(a) We used the NHANES 2013-14 hospital utilization and access to care questionnaire to obtain the fraction of individuals in each age group who did not have a healthcare visit for over 2 years (~Dx). We then used the NHANES 2013-14 BP and cholesterol questionnaire to obtain

the fraction of those who had a healthcare visit in the last 2 years and were ever told they had high BP (DxHT).

(b) We used NHANES 2013-14 to estimate the true prevalence of hypertension based on actual BP readings among individuals not taking anti-hypertension medication. Estimates of sensitivity and specificity for BP measurement from SMBP and CBPM were obtained from estimates reported by Lovibond, et al (1) based on a previous meta-analysis of Hodgkinson, et al (11). These values were used to obtain positive and negative predicted values under SMBP and CBPM diagnosis, which were adjusted for the National Health and Nutrition Examination Survey (NHANES), 2013-2014 hypertension prevalence rate (12). For those diagnosed as normotensive in the model (DxNT), we calculated the positive and negative predictive values as retesting after a first diagnosis based on usual care.

(c) True prevalence and sensitivity and specificity for each technology were also used to determine diagnosis of hypertension.

(d) We used NHANES 2013-14 to estimate the fraction of individuals with hypertension that are adherent to their medication.

Table B. CBPM and SMBP parameters

Input Variable	CBPM Parameters	SMBP Parameter	Reference
(a) Probabilities, annual [i]			
Probability of no diagnosis	0.8-11.6%	0.8-11.6%	NHANES 2013-14 (12)
Probability of HT diagnosis	16.2-83.8%	16.2-83.8%	NHANES 2013-14 (12)
(b) and (c) Diagnosis accuracy			
Sensitivity	85.6%	85.7%	Lovibond et al. (1)
Specificity	45.9%	62.4%	Lovibond et al. (1)
(d) Hypertension management			
Adherence therapeutic treatment [i]	30.7–89.6%	31.7-90.3%	NHANES 2013-14 (12) and Agarwal et al. (8)

[i] Age-dependent. List probabilities from different age groups, from 25-34 to 85+ years old.

3.2 Part 2: Markov model

Following the annotations in Fig B., the following parameters were assumed in the model.

(g) We used the 10-year Framingham risk equation to evaluate the average patient from the simulated cohort. Annual probabilities of CVD events are obtained from the current age of the cohort until age 100. All surviving individuals are assumed to die at age 100. One-year probabilities of CVD events are obtained separately for males and females for each CVD event (MI, stroke and other CVD) based on the 2008-2010 average of CVD distribution by age. Probabilities are then combined based on female-male distribution. Age-dependent probabilities are presented in Table C. For illustration, one-year probabilities of CVD events for the age group 45 to 54 years old are represented in Fig C.

Fig C. One-year probabilities of CVD events for age group 45-54 (SMBP, under treatment)

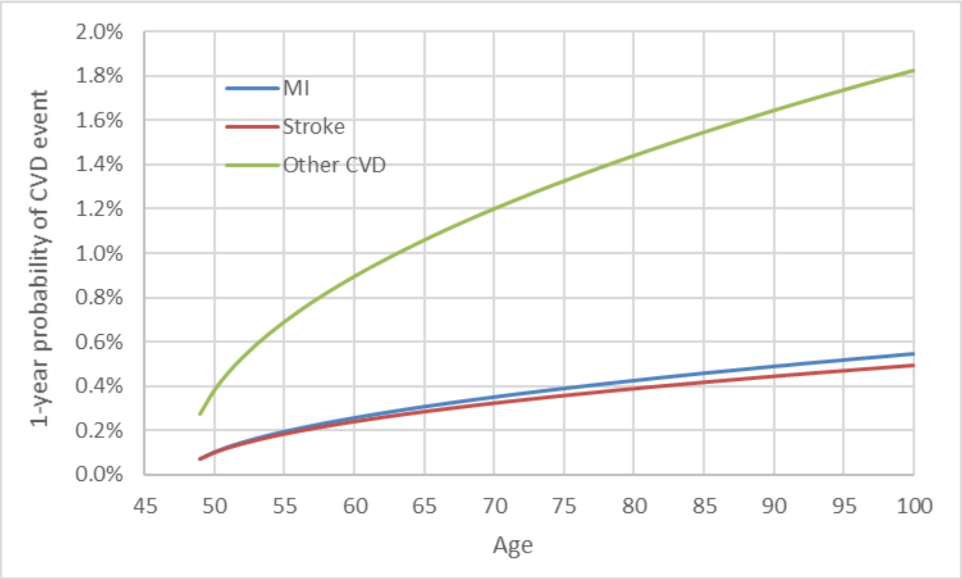

(i) Transition from CVD to post-CVD state occurs after one year for all surviving individuals.

(k) All surviving individuals remain in the post-CVD state for up to five years unless they have a subsequent CVD event, die, or leave the insurance plan.

(l) One-year probabilities of subsequent CVD events were obtained from 4-year follow-up Framingham data on subsequent CVD events (13). One-year probabilities of CVD events were obtained separately for males and females for each CVD event (MI, stroke and other CVD) based on the 2008-2010 average of CVD distribution by age. Probabilities were then combined based on female-male distribution. Age-dependent probabilities are presented in Table C. Fig D shows the 1-year probabilities of subsequent CVD events for the age group 45 to 54 years old.

Fig D. One-year probabilities of subsequent CVD events for age group 45-54 (SMBP)

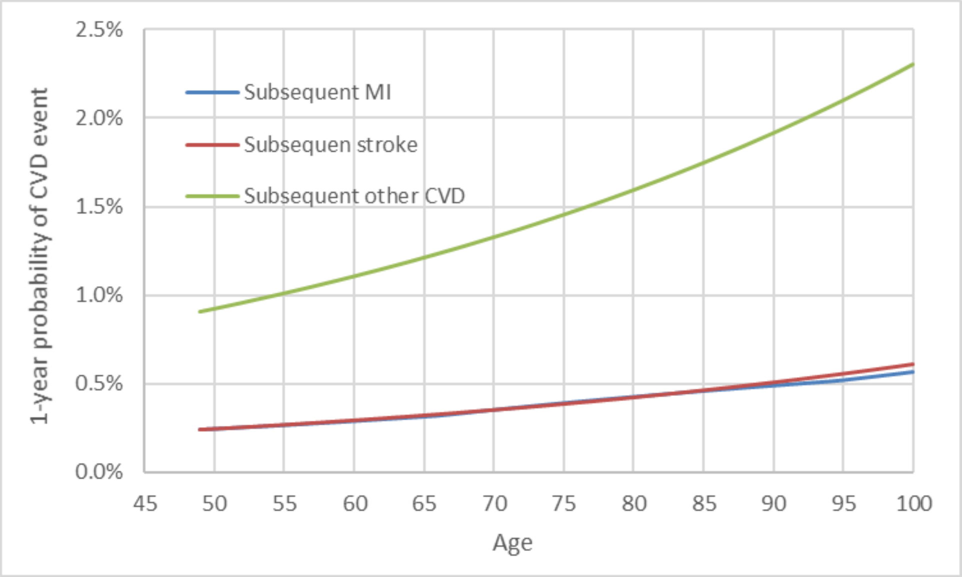

(m) Return to baseline risk is assumed after 5 years of cardiovascular disease (calculated).

(n) Transition to death (absorbing state) assumes a non-CVD mortality rate of 0.5-18.3% (age-dependent). These were obtained from the 2010 U.S. life tables(14). CVD mortality rates at first year (after MI, stroke or other CVD) were 6.3-100% (age-dependent)(15, 16). CVD mortality at second-to-fifth year were 2.9-67.3% (age- and time-dependent)(15, 16). We used the Truven database to estimate turnover rates for individuals leaving the insurance plan. A random sample of 1000 individuals in each age category (25-34, 35-44, 45-54, 55-64) enrolled in a commercial plan as of January 2010 were pulled from the Truven database. The cohort was followed to assess whether they were still enrolled at one year (January 2011) to five years (January 2015) later. The turnover rate for each age group was calculated as the average rate of individuals leaving the plan each year over the 5-year-period. Results of the analysis are presented in Table C.

Table C. Probabilities

Input Variable	Parameter CBPM	Parameter SMBP	Reference
(g) Probabilities, annual [i]			
Incident MI			
Age 25-34	0.012-0.094%	0.011-0.09%	D’Agostino et al. (17)
Age 35-44	0.027-0.202%	0.026-0.193%	D’Agostino et al. (17)
Age 45-54	0.076-0.57%	0.073-0.545%	D’Agostino et al. (17)

Age 55-64	0.204-1.359%	0.195-1.302%	D'Agostino et al. (17)
Age 65-74	0.306-1.724%	0.292-1.653%	D'Agostino et al. (17)
Age 75-84	0.485-2.305%	0.465-2.215%	D'Agostino et al. (17)
Age 85+	0.487-1.452%	0.464-1.387%	D'Agostino et al. (17)
Incident stroke			
Age 25-34	0.013-0.104%	0.013-0.1%	D'Agostino et al. (17)
Age 35-44	0.031-0.224%	0.029-0.214%	D'Agostino et al. (17)
Age 45-54	0.076-0.516%	0.072-0.492%	D'Agostino et al. (17)
Age 55-64	0.147-0.914%	0.141-0.874%	D'Agostino et al. (17)
Age 65-74	0.225-1.191%	0.215-1.14%	D'Agostino et al. (17)
Age 75-84	0.36-1.622%	0.345-1.556%	D'Agostino et al. (17)
Age 85+	0.446-1.304%	0.425-1.245%	D'Agostino et al. (17)
Incident other CVD			
Age 25-34	0.075-0.585%	0.071-0.558%	D'Agostino et al. (17)
Age 35-44	0.172-1.255%	0.163-1.196%	D'Agostino et al. (17)
Age 45-54	0.284-1.915%	0.27-1.826%	D'Agostino et al. (17)
Age 55-64	0.418-2.515%	0.398-2.403%	D'Agostino et al. (17)
Age 65-74	0.59-3.083%	0.562-2.95%	D'Agostino et al. (17)
Age 75-84	0.833-3.794%	0.797-3.641%	D'Agostino et al. (17)
Age 85+	0.965-2.834%	0.919-2.706%	D'Agostino et al. (17)
(I) Probabilities, annual [i]			
Subsequent MI			
Age 25-34	0.109-0.378%	0.109-0.376%	D'Agostino et al. (13)
Age 35-44	0.137-0.399%	0.136-0.396%	D'Agostino et al. (13)

Age 45-54	0.24-0.57%	0.239-0.566%	D'Agostino et al. (13)
Age 55-64	0.438-1.359%	0.435-1.302%	D'Agostino et al. (13)
Age 65-74	0.534-1.724%	0.529-1.653%	D'Agostino et al. (13)
Age 75-84	0.674-2.305%	0.668-2.215%	D'Agostino et al. (13)
Age 85+	0.74-1.452%	0.728-1.387%	D'Agostino et al. (13)
Subsequent stroke			
Age 25-34	0.122-0.425%	0.121-0.423%	D'Agostino et al. (13)
Age 35-44	0.153-0.451%	0.152-0.447%	D'Agostino et al. (13)
Age 45-54	0.244-0.618%	0.242-0.612%	D'Agostino et al. (13)
Age 55-64	0.331-0.914%	0.327-0.874%	D'Agostino et al. (13)
Age 65-74	0.416-1.191%	0.411-1.14%	D'Agostino et al. (13)
Age 75-84	0.539-1.622%	0.532-1.556%	D'Agostino et al. (13)
Age 85+	0.7-1.304%	0.688-1.245%	D'Agostino et al. (13)
Subsequent other CVD			
Age 25-34	0.682-2.388%	0.68-2.377%	D'Agostino et al. (13)
Age 35-44	0.86-2.534%	0.854-2.516%	D'Agostino et al. (13)
Age 45-54	0.914-2.327%	0.906-2.303%	D'Agostino et al. (13)
Age 55-64	0.953-2.515%	0.942-2.403%	D'Agostino et al. (13)
Age 65-74	1.101-3.083%	1.086-2.95%	D'Agostino et al. (13)
Age 75-84	1.229-3.794%	1.214-3.641%	D'Agostino et al. (13)
Age 85+	1.503-2.834%	1.478-2.706%	D'Agostino et al. (13)
(n) Mortality rates and turnover rates, annual [i]			
Post-incident MI (year 1)			
Age 25-34	3.355-100%	Same as CBPM	CDC [(14)], Bronnun et al. (16)

Age 35-44	3.879-100%	Same as CBPM	CDC [14], Bronnun et al. (16)
Age 45-54	5.586-100%	Same as CBPM	CDC [14], Bronnun et al. (16)
Age 55-64	9.014-100%	Same as CBPM	CDC [14], Bronnun et al. (16)
Age 65-74	10.003-100%	Same as CBPM	CDC [14], Bronnun et al. (16)
Age 75-84	20.885-100%	Same as CBPM	CDC [14], Bronnun et al. (16)
Age 85+	68.744-100%	Same as CBPM	CDC [14], Bronnun et al. (16)
Post-incident stroke (year 1)			
Age 25-34	2.435-100%	Same as CBPM	CDC [14], Bronnun et al. (15)
Age 35-44	2.816-100%	Same as CBPM	CDC [14], Bronnun et al. (15)
Age 45-54	4.055-100%	Same as CBPM	CDC [14], Bronnun et al. (15)
Age 55-64	6.544-100%	Same as CBPM	CDC [14], Bronnun et al. (15)
Age 65-74	12.163-100%	Same as CBPM	CDC [14], Bronnun et al. (15)
Age 75-84	19.801-100%	Same as CBPM	CDC [14], Bronnun et al. (15)
Age 85+	65.175-100%	Same as CBPM	CDC [14], Bronnun et al. (15)
Post-other CVD (year 1)			
Age 25-34	2.895-100%	Same as CBPM	Authors' estimation
Age 35-44	3.347-100%	Same as CBPM	Authors' estimation
Age 45-54	4.821-100%	Same as CBPM	Authors' estimation
Age 55-64	7.779-100%	Same as CBPM	Authors' estimation
Age 65-74	11.083-100%	Same as CBPM	Authors' estimation
Age 75-84	20.343-100%	Same as CBPM	Authors' estimation
Age 85+	66.959-100%	Same as CBPM	Authors' estimation
Post-incident MI (years 2–5)			
Age 25-34	1.464-67.276%	Same as CBPM	CDC [14], Bronnun et al. (16)

Age 35-44	1.693-67.276%	Same as CBPM	CDC [14], Bronnun et al. (16)
Age 45-54	2.438-67.276%	Same as CBPM	CDC [14], Bronnun et al. (16)
Age 55-64	3.934-67.276%	Same as CBPM	CDC [14], Bronnun et al. (16)
Age 65-74	4.674-67.276%	Same as CBPM	CDC [14], Bronnun et al. (16)
Age 75-84	9.757-67.276%	Same as CBPM	CDC [14], Bronnun et al. (16)
Age 85+	32.118-67.276%	Same as CBPM	CDC [14], Bronnun et al. (16)
Post-incident stroke (years 2–5)			
Age 25-34	1.337-60.914%	Same as CBPM	CDC [14], Bronnun et al. (15)
Age 35-44	1.546-60.914%	Same as CBPM	CDC [14], Bronnun et al. (15)
Age 45-54	2.226-60.914%	Same as CBPM	CDC [14], Bronnun et al. (15)
Age 55-64	3.592-60.914%	Same as CBPM	CDC [14], Bronnun et al. (15)
Age 65-74	6.677-60.914%	Same as CBPM	CDC [14], Bronnun et al. (15)
Age 75-84	8.835-60.914%	Same as CBPM	CDC [14], Bronnun et al. (15)
Age 85+	29.08-60.914%	Same as CBPM	CDC [14], Bronnun et al. (15)
Post-other CVD (years 2–5)			
Age 25-34	1.4-64.095%	Same as CBPM	Authors’ estimation
Age 35-44	1.619-64.095%	Same as CBPM	Authors’ estimation
Age 45-54	2.332-64.095%	Same as CBPM	Authors’ estimation
Age 55-64	3.763-64.095%	Same as CBPM	Authors’ estimation
Age 65-74	5.675-64.095%	Same as CBPM	Authors’ estimation
Age 75-84	9.296-64.095%	Same as CBPM	Authors’ estimation
Age 85+	30.599-64.095%	Same as CBPM	Authors’ estimation
Non CVD related			
Age 25-34	0.085-18.305%	Same as CBPM	CDC [14]

Age 35-44	0.142-18.305%	Same as CBPM	CDC [14]
Age 45-54	0.293-18.305%	Same as CBPM	CDC [14]
Age 55-64	0.605-18.305%	Same as CBPM	CDC [14]
Age 65-74	1.314-18.305%	Same as CBPM	CDC [14]
Age 75-84	2.809-18.305%	Same as CBPM	CDC [14]
Age 85+	8.636-18.305%	Same as CBPM	CDC [14]
Insurance turnover [i]			
Age 25-34	16.623-18.19%	Same as CBPM	Authors' estimation
Age 35-44	20.338-18.19%	Same as CBPM	Authors' estimation
Age 45-54	20.483-18.19%	Same as CBPM	Authors' estimation
Age 55-64	18.19-18.19%	Same as CBPM	Authors' estimation

[i] Time-dependent. Probabilities change over time as the cohort ages. List probabilities from the average age of the cohort until 100 years old. For mortality rates the list is until 99 years old because all surviving individuals are assumed to die at age 100.

4. Treatment and CVD costs

Following the annotations in Figs A and B, the following costs were assumed in the model.

(c) For the SMBP intervention, we assume an initial investment in the device, priced at a retail cost of $50, and including additional educational support to the patient paid by insurance (see Table C). When SMBP is used to diagnose and select treatment (Use Cases #1 and #2), a 30-minute nurse-provided training session valued at $34.70/hour (18) is included. When SMBP is used for ongoing BP monitoring (Use Case #3), we included one-hour of nurse training annually for educational support to the patient. We also assumed that all patients with hypertension receive an SMBP device for ongoing BP monitoring that is replaced every 5 years. In all use cases, we assumed patients kept the SMBP device and that copayments were not required.

(d) As described in section 2 of this Appendix, we used an episode-of-care approach to characterize the interval encompassing diagnosis and anti-hypertensive treatment selection and dosage titration. We identified all costs associated with a hypertension-related episode of care, including laboratory tests, doctor's visits, and hospital and emergency department visits incurred during the identified episode. Since CBPM, but not SMBP, is still used extensively in the United States to diagnose hypertension, we assumed that cost estimates based on Truven

database statistics were representative of usual care (CBPM). For SMBP, potential reductions in utilization were estimated as follows. We assumed that those having 4 or more visits to reach a treatment selection under usual care, will have one fewer visit under SMBP. Using the Truven database, we estimated the average cost of the last visit saved for those with 4 or more visits at $650.6. This estimate translates to an average cost per person of SMBP-based diagnosis and treatment selection of $3,014, compared to $3,159 of CBPM (see Table D).

(f) Baseline cost for management of hypertension was estimated at $941/year when individuals are adherent to medication, and $567/year when they are not. These values are from MEPS(19) and corresponded to the mean expenditure per hypertensive adult among those receiving care for hypertension in 2010. We subtracted mean expenditure in prescription medications to assess management cost of non-adherent individuals. Costs were inflated to 2019 dollars using the medical inflation rate.

(h) CVD event states were myocardial infarction (MI), stroke and other CVD disease events (Other) including unstable angina and transient ischemic attack. First-year costs were: MI at $57,243/year, stroke at $47,997/year, and other CVD at $22,156/year(20). Costs were inflated to 2019 dollars using the medical inflation rate.

(j) For the post-CVD states, we considered second-year cost of $11,162/year, $14,322/year and $5,630/year for MI, stroke and other CVD, respectively(20). Costs converge to baseline in year 5. Costs were inflated to 2019 dollars using the medical inflation rate.

Table D. Costs

Input Variable	Parameter	Uncertainty Range	Reference
(c) Intervention cost (2019 dollars)			
SMBP device	$50	NU	Authors' estimation (average retail price)
SMBP co-payment	$0	NU	
Education support-SMBP diagnosis	$17.4	NU	BLS (0.5 hours/year, RN wage)(18)
SMBP device replacement (years)	5	NU	Authors' estimation (average equipment life)
Education support-SMBP management	$34.7		BLS (1 hour/year, RN wage)(18)
(d) Hypertension diagnosis and treatment selection cost (2019 dollars)			
CBPM diagnosis & treatment selection	$3,159	NU	Authors' estimation
SMBP diagnosis & treatment selection	$3,014	NU	Authors' estimation
(f) Hypertension management cost (2019 dollars), annual			
Adherent to medication (Tx)	941	705–1,176	MEPS (19)
No adherent to medication (~Tx)	567	425–709	MEPS (19)
(h) Cost of CVD events (2019 dollars), annual			

MI, acute	60,365	46,150–76,916	Bonafede et al (20)
Stroke, acute	51,593	38,695–64,492	Bonafede et al (20)
Other CVD, acute	26,103	17,862–29,770	Bonafede et al (20)
(i) Cost of post-CVD events			
MI, chronic at 2 years	12,218	8,999–14,999	Bonafede et al (20)
Stroke, chronic at 2 years	18,667	11,547–19,244	Bonafede et al (20)
Other CVD, chronic at 2 years	6,173	4,539–7,565	Bonafede et al (20)

NU: No uncertainty included.
SE: Standard error.

5. Payer's benefits

From a commercial insurance perspective, the payer receives a premium revenue stream from all surviving members who remain in the insurance plan.

(o) Exiting the model (death or leaving the insurance plan) represents an elimination of the premium revenue stream. Average premiums were obtained from an average commercial insurance plan from eHealth website(21) and from the average Medicare advantage plan from CMS(22). Parameters are reported in Table E. Note that premium gains are independent of the cohort health status, and they are only affected by permanence in the plan. Since type of intervention does not affect insurance turnover rate, the only effect of SMBP on insurance premium savings is through its indirect effect on CVD mortality. Finally, commercial insurance companies reduce their financial burden through patient cost-sharing. In our model, we assume annual deductibles for health care services, as shown in Table E.

Table E. Insurance parameters

Input Variable	Parameter	Uncertainty Range	Reference
(o) Health insurance parameters			
Premiums ($)			
Age 25-64	223-520	Age-dependent	eHealth (21)
Age > 65	835	752–1,044	CMS
Deductibles ($)			
Age 25-64	3,616-3,882	Age-dependent	eHealth(21)
Age > 65	2,158	1,942–2,698	Kaiser Family Foundation(23)

6. Sensitivity analysis

We performed sensitivity analyses to assess how the NPV of the SMBP investment changes with variations in the three SMBP use cases. With respect to diagnosis (Use Case #1), SMBP is known to have better diagnostic specificity than CBPM, but similar sensitivity (11). This enables SMBP to detect the white coat hypertension (WCH) and avoid false positive diagnoses. Results in Table F show that an investment in SMBP monitoring is sensitive to the accuracy of SMBP measurement in detecting WCH. A 10% reduction in SMBP specificity reduces the investment's NPV by more than 45%, with higher reductions among older age groups.

Table F. Change in NPV when diagnosis accuracy changes

Age Group	Baseline NPV	Effect of specificity on NPV (baseline specificity rate: 62.4%)*	
		To 56.2% specificity rate (10% reduction)	To 68.6% specificity rate (10% increase)
Age 25-34	$442	-$203 (-45.9%)	$202 (45.7%)
Age 35-44	$379	-$177 (-46.7%)	$176 (46.5%)
Age 45-54	$309	-$154 (-49.9%)	$155 (50.1%)
Age 55-64	$135	-$100 (-74.4%)	$101 (74.5%)
Age 65-74	$56	-$78 (-138.9%)	$78 (139%)
Age 75-84	$16	-$49 (-303.9%)	$49 (304.1%)
Age 85+	-$70	-$9 (-12.8%)	$9 (12.7%)
**Total Per Individual**	**$254**	**-$136 (-53.5%)**	**$136 (53.4%)**

*Change in NPV (in 2019 dollars). In parenthesis: change relative to baseline NPV in absolute value (in percentages)

For treatment selection (Use Case #2), we assume that physicians using CBPM require more visits to select and titrate treatment. Sensitivity analysis were performed to assess the effect of saving 2 visits with SMBP among those who had 4 or more visits with CBPM. We also assessed the effect of saving 1 visit among those with 3 or more visits, and among those with 5 or more visits. Results presented in Table G show a moderate impact on NPV, except for individuals aged 65 or older.

Table G. Change in NPV when visits for treatment selection change

Age Group	Baseline	Effect of fewer visits for treatment selection on NPV (baseline: 1 visit saved among those with 4+ visits)*		
		To 2 visits saved among those with 4+ visits	To 1 visit saved among those with 3+ visits	To 1 visit saved among those with 5+ visits
Age 25-34	$442	-$32 (-7.3%)	-$29 (-6.6%)	-$15 (-3.5%)
Age 35-44	$379	-$30 (-7.8%)	-$27 (-7.1%)	-$14 (-3.7%)
Age 45-54	$309	-$25 (-8%)	-$23 (-7.3%)	-$11 (-3.6%)
Age 55-64	$135	-$18 (-13.7%)	-$17 (-12.5%)	-$8 (-6.2%)
Age 65-74	$56	-$16 (-28.7%)	-$15 (-26.2%)	-$-7 (13.1%)
Age 75-84	$16	-$33 (-208.6%)	-$30 (-190.2%)	-$15 (-95%)
Age 85+	-$70	-$-5 (-7.6%)	-$-5 (-7%)	-$-2 (-3.5%)
**Total Per Individual**	**$254**	**-$23 (-9.1%)**	**-$21 (-8.3%)**	**-$11 (-4.2%)**

*Change in NPV (in 2019 dollars). In parenthesis: change relative to baseline NPV in absolute value (in percentages)

For the ongoing BP monitoring (Use Case #3), we assume that SMBP improves the patient's medication adherence rate, which translates into more hypertensive patients (whether diagnosed by CMBP or SMBP) receiving treatment. We further assume that the use of SMBP monitoring reduces BP levels, with a reduced CVD risk in our Markov model. A sensitivity analysis was performed for both parameters (adherence to antihypertension medication and reduction in systolic BP). Results are presented in Table H.

Table H. Change in NPV when management of hypertension changes

Age Group	Baseline	Change in Medication Adherence (baseline: 0.21 s.m. difference)*		Reduction in Systolic BP (baseline: 2.63mmHg difference)*	
		To 0.23 s.m. difference (10% increase)	To 0.19 s.m. difference (10% reduction)	To 2.37 mmHg (10% reduction)	To 2.89 mmHg (10% increase)
Age 25-34	$442	$0 (0%)	-$1 (-0.2%)	-$1 (-0.1%)	$1 (0.1%)
Age 35-44	$379	$0 (0.1%)	-$1 (-0.2%)	-$0 (-0.1%)	$0 (0.1%)
Age 45-54	$309	$1 (0.2%)	-$0 (-0.1%)	-$0 (-0.1%)	$0 (0.1%)
Age 55-64	$135	$1 (0.6%)	-$1 (-0.5%)	-$0 (-0.1%)	$0 (0.2%)
Age 65-74	$56	$0 (1%)	-$0 (-0.9%)	-$0 (-0.8%)	$0 (0.9%)
Age 75-84	$16	$1 (13.7%)	-$1 (-13.4%)	-$0 (-8.4%)	$1 (8.6%)
Age 85+	-$70	$1 (0.8%)	-$1 (-0.8%)	-$0 (-0.1%)	$0 (0%)
**Total Per Individual**	**$254**	$0 (0.2%)	-$1 (-0.3%)	-$0 (-0.1%)	$0 (0%)

*Change in NPV (in 2019 dollars). In parenthesis: change relative to baseline NPV in absolute value (in percentages)

The standardized mean (s.m.) difference associated with greater medication adherence due to SMBP use is estimated at 0.21(9), which represents an increment of nearly 1 percent in the percentage of individuals adherent to treatment compared to usual care. For example, for the age group 25-34 the percentage of individual adherent to medication treatment is estimated at 30.7% with an standard deviation of 4.4% based on NHANES(12) data. That puts the adherence rate under usual care at 30.7% and under SMBP at 31.7% (30.7% − 0.21 x 4.4%). For the sensitivity analysis we assessed the effect of a 10% increase and a 10% reduction in the baseline s.m. difference in medication adherence. Table H shows that the effect on NPV is not sensitive to changes in adherence, except for age group 75-84. A similar result is observed for the reduction in systolic BP associated to SMBP use. The impact on NPV is negligible for non-elderly adults.

7. Uncertainty analysis

We performed Monte Carlo simulations with 1,000 draws to account for uncertainty in parameters (probabilities, rates, costs and insurance cost sharing). For each age-group, we obtained the probability of a favorable NPV, assessed as the fraction of simulated Monte Carlo draws with a positive NPV. Table I shows the NPV distribution for each age group, indicating a more disperse distribution for older cohorts.

Table I. Simulated probability distribution of NPV

NPV	Age 25-34	Age 35-44	Age 45-54	Age 55-64	Age 65-74	Age 75-84	Age 85+
NPV≤-4500	0%	0%	0%	0%	0%	0%	0%
-4500<NPV≤-4000	0%	0%	0%	0%	0%	0%	0%
-4000<NPV≤-3500	0%	0%	0%	1%	0%	0%	0%
-3500<NPV≤-3000	0%	0%	0%	0%	0%	0%	0%
-3000<NPV≤-2500	0%	0%	0%	1%	1%	1%	0%
-2500<NPV≤-2000	0%	0%	0%	1%	2%	1%	0%
-2000<NPV≤-1500	0%	0%	0%	1%	2%	3%	0%
-1500<NPV≤-1000	0%	0%	0%	3%	5%	5%	0%
-1000<NPV≤-500	0%	0%	0%	6%	7%	8%	0%
-500<NPV≤0	0%	2%	6%	15%	23%	27%	91%
0<NPV≤500	50%	90%	86%	53%	41%	41%	9%
500<NPV≤1000	50%	8%	7%	10%	9%	9%	0%
1000<NPV≤1500	0%	1%	1%	5%	5%	4%	0%
1500<NPV≤2000	0%	0%	0%	3%	3%	2%	0%
2000<NPV≤2500	0%	0%	0%	1%	1%	1%	0%
2500<NPV≤3000	0%	0%	0%	1%	1%	0%	0%
3000<NPV≤3500	0%	0%	0%	0%	0%	0%	0%
3500<NPV≤4000	0%	0%	0%	0%	0%	0%	0%
4000<NPV≤4500	0%	0%	0%	0%	0%	0%	0%
NPV>4500	0%	0%	0%	0%	0%	0%	0%

Fig E presents the probability of a favorable overall NPV (NPV>0) by age group. We also show probabilities of a favorable NPV broken down by use case when SMBP is used only for diagnosis, only for treatment selection, or only for ongoing hypertension management. Results coincide with our main finding that diagnosis and treatment selection (Use Cases #1 and 2) drive the positive NPV of the SMBP investment. When SMBP is used only for BP monitoring (Use Case #3), the investment produces negative NPVs in most cases.

Fig E. Probability of Positive NPV of SMBP Investment by Use Case and Age Group

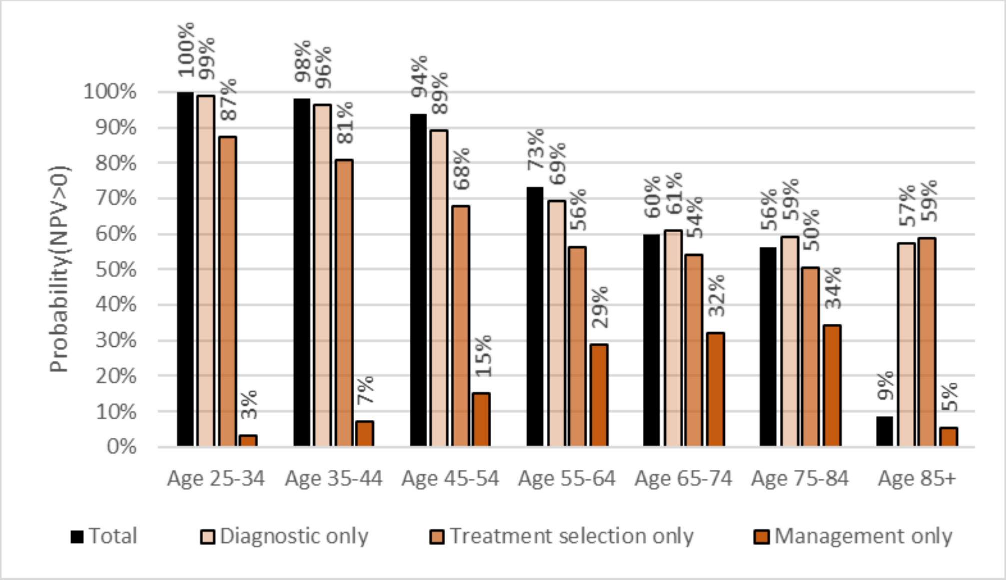

References

1. Lovibond K, Jowett S, Barton P, et al. Cost-effectiveness of options for the diagnosis of high blood pressure in primary care: a modelling study. Lancet 2011;378:1219-1230
2. Pickering TG, Coats A, Mallion JM, et al. Blood Pressure Monitoring. Task force V: White-coat hypertension. Blood Press Monit 1999;4:333-341
3. Merai R SC, Rakotz M, et al. CDC Grand Rounds: A Public Health Approach to Detect and Control Hypertension. MMWR Morb Mortal Wkly Rep 2016. 2016:1261–1264
4. Mozaffarian D BE, Go AS, Arnett DK, Blaha MJ, et al. Heart disease and stroke statistics—2015 update: a report from the American Heart Association. Circulation 2015;131:e29–e322
5. Ayman D GA. Blood pressure determination by patients with hypertension. In: The difference between clinic and home readings before treatment. American Journal of Medical Sciences 1940;200:465-474
6. Pickering TG, Eguchi K, Kario K. Masked hypertension: a review. Hypertens Res 2007;30:479-488
7. Muntner P, Shimbo D, Carey RM, et al. Measurement of Blood Pressure in Humans: A Scientific Statement From the American Heart Association. Hypertension 2019;73:e35-e66

8. Agarwal R, Bills JE, Hecht TJ, et al. Role of home blood pressure monitoring in overcoming therapeutic inertia and improving hypertension control: a systematic review and meta-analysis. Hypertension 2011;57:29-38
9. Fletcher BR, Hartmann-Boyce J, Hinton L, et al. The effect of self-monitoring of blood pressure on medication adherence and lifestyle factors: a systematic review and meta-analysis. American journal of hypertension 2015;28:1209-1221
10. Hansen L. The Truven health MarketScan databases for life sciences researchers. Truven Health Ananlytics IBM Watson Health2017
11. Hodgkinson J, Mant J, Martin U, et al. Relative effectiveness of clinic and home blood pressure monitoring compared with ambulatory blood pressure monitoring in diagnosis of hypertension: systematic review. BMJ 2011;342:d3621
12. National Center for Health Statistics. National Health and Nutrition Examination Survey, 2013–2014. Hyattsville: Centers for Disease Control and Prevention, US Department of Health and Human Services. 2013
13. D'Agostino RB, Russell MW, Huse DM, et al. Primary and subsequent coronary risk appraisal: new results from the Framingham study. American heart journal 2000;139:272-281
14. Centers for Disease Control and Prevention. Health Data Interactive. Available at: ftp://ftp.cdc.gov/pub/Health_Statistics/NCHS/Publications/NVSR/63_07/Table01.xlsx. Accessed March 7, 2017,
15. Bronnum-Hansen H, Davidsen M, Thorvaldsen P. Long-term survival and causes of death after stroke. Stroke 2001;32:2131-2136
16. Bronnum-Hansen H, Jorgensen T, Davidsen M, et al. Survival and cause of death after myocardial infarction: the Danish MONICA study. J Clin Epidemiol 2001;54:1244-1250
17. D'Agostino RB VR, Pencina MJ, Wolf PA, Cobain M, et al. . General cardiovascular risk profile for use in primary care: the Framingham Heart Study. Circulation 2008;117:743-753
18. Bureau of Labor Statistics. Occupational Employment and Wages, May 2016. 29-1141 Registered Nurses. Available at: https://www.bls.gov/oes/2016/may/oes291141.htm. Accessed November 12, 2017,
19. Davis KE. STATISTICAL BRIEF# 404: Expenditures for Hypertension among Adults Age 18 and Older, 2010: Estimates for the US Civilian Noninstitutionalized Population. AHRQ Agency for healthcare Research and Quality April 2013
20. Bonafede MM, Johnson BH, Richhariya A, et al. Medical costs associated with cardiovascular events among high-risk patients with hyperlipidemia. ClinicoEconomics and outcomes research: CEOR 2015;7:337
21. eHealth. eHealth Price Index. Available at: https://www.ehealthinsurance.com/resource-center/ehealth-price-index. Accessed March 7, 2017,
22. Centers for Medicare and Medicaid Services. Announcement of Calendar Year (CY) 2015 Medicare Advantage Capitation Rates and Medicare Advantage and Part D Payment Policies and Final Call Letter. Available at: https://www.cms.gov/Medicare/Health-Plans/MedicareAdvtgSpecRateStats/downloads/Announcement2015.pdf. Accessed March 7, 2017,
23. Kaiser Family Foundation. Medicare Part D: A First Look at Plan Offerings in 2014. Available at: http://kff.org/medicare/issue-brief/medicare-part-d-a-first-look-at-plan-offerings-in-2014/. Accessed March 7, 2017,
